# Adaptive Dual Reinforcement Learning for Hybrid Spatial–Temporal Networks in RIS-Assisted Indoor Localization (ADRL-HSTNet)

**DOI:** 10.3390/s26092890

**Published:** 2026-05-05

**Authors:** Mostafa Mohamed, Ahmed Radi, Shady Zahran

**Affiliations:** 1 Mobile Multi-Sensor Systems (MMSS) Research Group, Calgary, AB T2N 1N4, Canada; mostafa.mostafa@alumni.ucalgary.ca (M.M.); ahmed.elboraee@alumni.ucalgary.ca (A.R.); 2 Technical Research Center, Cairo 11477, Egypt

**Keywords:** reconfigurable intelligent surfaces (RISs), indoor localization, spatial–temporal modeling, reinforcement learning

## Abstract

Reconfigurable intelligent surface sensors (RISs) have emerged as a promising technology for enhancing wireless indoor localization by intelligently controlling signal propagation; however, extracting reliable localization fingerprints from RIS-assisted signals remains challenging due to multipath fading, environmental noise, and nonlinear spatial–temporal channel dynamics. To address this, we propose an Adaptive Dual-Reinforcement Learning-Hybrid Spatial–Temporal Network (ADRL-HSTNet) for RIS-assisted indoor localization. The framework utilizes dual-channel RSSI and phase measurements, followed by noise filtering, normalization, and sliding-window segmentation prior to feature extraction. It then constructs enhanced representations through handcrafted feature extraction and multi-branch processing, including patch-based features, wavelet-domain representations, statistical descriptors, and multi-level segmentation masks. These heterogeneous inputs are encoded using lightweight transformer-based encoders to capture multiscale dependencies. A first reinforcement learning selector adaptively weights the most informative feature branches to produce a fused representation, which is further processed by spatial and temporal transformer modules. Their outputs are adaptively combined via a second reinforcement learning selector to obtain robust localization embedding. The model jointly performs classification, coordinate regression, and uncertainty estimation end-to-end. Experimental results across multiple RIS configurations outperformed the KAN, LSTM-KAN, and RHL-Net (compared against the proposed ADRL-HSTNet) baselines, achieving accuracies of 83.33%, 75.22%, 93.33%, and 88.89%, confirming the effectiveness of the proposed approach.

## 1. Introduction

The rapid advancement of next-generation wireless communication systems, particularly fifth-generation (5G) and emerging sixth-generation (6G) networks, has intensified the demand for accurate localization across connected transportation, UAV operation, smart buildings, asset tracking, and immersive services [1]. Among these applications, indoor positioning remains especially challenging because global navigation satellite systems (GNSS) degrade severely in enclosed and dense environments due to blockage, multipath propagation, and non-line-of-sight (NLoS) conditions [2].

Indoor localization methods are generally divided into geometry-based and fingerprinting-based approaches. Geometry-based techniques, including time-of-arrival (ToA), time-difference-of-arrival (TDoA), and angle-of-arrival (AoA), can deliver high accuracy under favorable conditions, but they typically require precise synchronization and dedicated infrastructure. Fingerprinting methods based on received signal strength indicator (RSSI) or channel state information (CSI) are more flexible and cost-effective; however, their accuracy often deteriorates when the radio map is sparse, the environment changes, or the signal statistics drift over time.

Reconfigurable intelligent surfaces (RISs) have recently emerged as a powerful enabler for controllable radio environments. By adjusting the phase, amplitude, and reflection behavior, RISs can reshape propagation paths, improve coverage, and alleviate severe NLoS effects. These capabilities make RISs particularly attractive for localization, where stronger spatial diversity and more informative fingerprints can significantly improve positioning reliability.

In parallel, deep learning has shown strong potential for modeling the nonlinear and spatial–temporal behavior of wireless signals. Long short-term memory (LSTM) networks effectively capture temporal dependencies, while Kolmogorov–Arnold networks (KANs) provide flexible nonlinear function modeling. Recent RIS-aware deep learning work further indicates that combining advanced representation learning with structured signal modeling can substantially improve localization performance in complex indoor environments. Nevertheless, many existing methods still depend on fixed fusion rules or a limited set of feature domains, reducing their adaptability to diverse RIS configurations and environmental dynamics.

To address these limitations, this paper proposes an Adaptive Dual-Reinforcement Learning-Hybrid Spatial–Temporal Network (ADRL-HSTNet) for RIS-assisted indoor localization. The framework processes dual-channel RSSI and phase measurements through preprocessing, segmentation, handcrafted feature extraction, and multi-branch representation learning. Lightweight transformer encoders capture multiscale dependencies, while two reinforcement-learning selectors adaptively fuse heterogeneous branches and spatial–temporal embeddings. This design enables robust, uncertainty-aware localization and improves generalization across challenging indoor propagation conditions.

The main contributions of this work are summarized as follows:

1—Adaptive hybrid spatial–temporal architecture: A unified framework that integrates multi-branch feature representations with transformer-based spatial and temporal modeling to enhance localization performance in RIS-assisted environments.

2—Dual-stage reinforcement learning fusion: A novel adaptive selection mechanism that dynamically weights heterogeneous feature branches and spatial–temporal representations, improving feature integration and reducing redundancy.

3—Comprehensive multi-domain feature design: The incorporation of raw signals, wavelet features, statistical descriptors, segmentation masks, and handcrafted features to capture diverse signal characteristics.

4—Data-efficient generalization and fair evaluation: ADRL-HSTNet is trained and validated using only 70% of the available data, while 30% is reserved for testing. In contrast, the benchmark methods reported in [3]—KAN, LSTM-KAN, and RHL-Net—use 80% of the data for training/validation and only 20% for testing. Despite learning from 10% less data and being evaluated on 10% more unseen data, the proposed ADRL-HSTNet still achieves superior performance, highlighting stronger generalization and better data efficiency.

5—Robust performance improvement: Extensive experiments on USRP-collected datasets demonstrate that ADRL-HSTNet outperforms KAN, LSTM-KAN, and RHL-Net, achieving accuracies of 83.33%, 75.22%, 93.33%, and 88.89%, confirming its effectiveness in complex RIS-assisted environments.

The remainder of this paper is organized as follows. Section 2 reviews related work. Section 3 presents the proposed ADRL-HSTNet framework and methodology. Section 4 summarizes the hardware setup and implementation details. Section 5 provides the experimental results, ablation analysis, regression and uncertainty evaluation, and in-depth discussion. Finally, Section 6 concludes the paper.

## 2. Related Work

### 2.1. Conventional Geometry-Based and Fingerprinting Approaches

Conventional indoor localization research has historically been dominated by geometry-based and fingerprinting-based approaches. Early foundational works such as Farid et al. [4] established a comprehensive taxonomy of localization techniques, demonstrating that while range-based methods (e.g., ToA, TDoA, AoA) can theoretically achieve sub-meter accuracy, their performance is fundamentally constrained by synchronization requirements, line-of-sight assumptions, and severe degradation under multipath conditions. Despite providing critical system-level insights, these studies remain descriptive and do not offer adaptive mechanisms to address dynamic indoor environments.

Zafari et al. [5] extended this perspective by systematically analyzing deployment trade-offs, highlighting that RSSI-based approaches provide cost-effective and scalable solutions but suffer from significant sensitivity to environmental variations, user orientation, and signal drift. While this work clarifies practical limitations, it does not resolve the core issue of non-stationary signal behavior, which continues to limit robustness.

Fingerprinting-based methods, such as those reviewed in [6], introduced data-driven learning frameworks that improve localization performance under controlled conditions. However, these approaches remain heavily dependent on dense radio map construction and periodic recalibration, resulting in high maintenance cost and poor scalability [7]. More importantly, they lack adaptive feature selection mechanisms capable of responding to environmental changes.

Model-assisted approaches, including path-loss-based fingerprinting [8] and reconstructed databases [9], attempt to reduce the calibration effort. Although these methods improve practicality, their performance is fundamentally limited by propagation model mismatch. In realistic indoor environments, such assumptions often fail, leading to unstable performance and limited generalization.

Probabilistic and reinforcement-based frameworks [10,11,12] introduce improved scalability and partial adaptivity. Nevertheless, these approaches rely on predefined probabilistic structures or simplified decision models, and they do not fully exploit heterogeneous feature representations or incorporate uncertainty-aware learning. Consequently, their robustness under dynamic RIS-assisted environments remains limited.

### 2.2. Deep Learning and KAN-Based Localization

Deep learning approaches were introduced to overcome the limited expressiveness of traditional models. LSTM networks [13] provide strong temporal modeling capabilities by capturing sequential dependencies in wireless signals. However, their recurrent nature limits parallel processing and often underutilizes spatial correlations across subcarriers.

Kolmogorov–Arnold networks (KAN) [14] improve nonlinear function approximation by replacing fixed activation functions with learnable components. This enhances flexibility and interpretability; however, KAN alone does not explicitly model temporal dependencies or multi-domain feature interactions, limiting its effectiveness in complex localization scenarios.

Dahou et al. [3] extended KAN-based models to RIS-assisted localization, demonstrating improved performance through hybrid architectures such as LSTM-KAN and RHL-Net. While these models achieve competitive accuracy, their design remains constrained by limited feature diversity and shallow fusion strategies. In particular, they do not incorporate adaptive multi-branch selection or uncertainty-aware decision mechanisms.

Survey works such as [15,16,17] further confirm that deep learning improves localization performance but remains highly dependent on dataset size, environmental consistency, and feature design. These studies consistently highlight the lack of generalization and robustness across different environments.

### 2.3. Transformer and Adaptive Learning Approaches

Transformer-based models [18,19,20] have recently been applied to indoor localization due to their ability to capture long-range dependencies. These models achieve strong performance, particularly when processing heterogeneous telemetry data. However, they typically require large datasets and high computational resources, and they often rely on single-stream representations without adaptive feature selection.

RIS-aware adaptive frameworks [21,22] demonstrate that controlling signal propagation can significantly enhance localization performance. Nevertheless, these approaches primarily focus on system-level optimization rather than end-to-end learning, leaving the problem of adaptive feature fusion largely unaddressed.

### 2.4. RIS-Assisted Indoor Localization

RIS technology introduces a paradigm shift by enabling programmable propagation environments. Foundational works [23,24,25,26] establish the theoretical potential of RISs for improving signal diversity and coverage. However, these studies do not provide complete learning-based localization frameworks.

More advanced works [22,27,28,29,30,31,32] focus on estimation theory, beamforming, and calibration. While they achieve high theoretical accuracy, their reliance on idealized models limits applicability in real-world environments.

Recent experimental studies [33,34,35,36] demonstrate practical RIS benefits, achieving significant improvements in localization accuracy. However, these approaches typically rely on fixed feature representations and lack adaptive multi-domain fusion mechanisms, which limits robustness under varying conditions.

### 2.5. Research Gaps

The above literature reveals several gaps that remain insufficiently addressed in current RIS-assisted indoor localization research.

(i) Most existing methods still rely on a single dominant representation domain. Classical systems typically depend on RSSI, CSI, or timing features alone [8,9,10,11], while many recent deep models are built around one primary signal view or one sensor tokenization strategy [3,18,19,21]. Even when an RIS is used to enrich propagation [22,27,28,29,30,31,32,33,34,35,36], the downstream learner often exploits only a limited subset of the potentially available raw, spectral, statistical, phase, or segmentation-level cues. This weak feature diversity constrains robustness under changing RIS states, antenna patterns, and room geometries.

(ii) Fusion in the literature is usually static rather than adaptive. Conventional fingerprinting pipelines use deterministic matching rules; many learning-based approaches use fixed concatenation, averaging, or single-stream encoding; and several RIS-oriented estimators rely on predetermined signal models [3,27,28,29,30,31,32]. Such designs assume that all feature groups contribute equally across all samples, which is rarely true in practice. Indoor wireless signatures are highly condition-dependent, so an effective system should learn to upweight the most informative branches and suppress unreliable ones on a per-sample basis.

(iii) Spatial and temporal dependencies are frequently modeled in isolation instead of jointly and adaptively. Recurrent models emphasize sequential evolution [13], whereas many transformer or geometry-driven methods emphasize global token interactions or structural estimation [18,19,27,28,29,30,31]. However, RIS-assisted fingerprints are inherently hybrid: they contain spatial structure across subcarriers, antennas, and engineered feature maps while also exhibiting temporal fluctuation across symbols and observation windows. Treating these dependency types independently can leave complementary information underexploited.

(iv) Uncertainty awareness is still underdeveloped. Several studies have optimized accuracy, error bounds, or success rate [16,27,28,30,33,36], but comparatively few have integrated predictive confidence directly into representation selection and final decision formation [37]. This matters because branch reliability can change from one sample to another due to noise, blockage, hardware variation, or unfavorable RIS states. Without uncertainty-aware selection, the model may confidently fuse low-quality evidence and propagate avoidable errors.

Motivated by the identified research gaps, the proposed ADRL-HSTNet extends the conventional RIS-assisted fingerprinting paradigm through three key innovations. First, it introduces a rich multi-branch representation framework that captures complementary signal characteristics across the spatial, spectral, statistical, and segmentation domains, thereby enhancing feature diversity and robustness. Second, it employs a dual-stage adaptive reinforcement learning mechanism, where an initial selector performs dynamic branch-level fusion, followed by a second selector that adaptively integrates spatial and temporal representations, enabling context-aware decision making. Third, the framework incorporates uncertainty-aware learning, allowing the model to estimate the reliability of intermediate representations and final predictions, thereby improving robustness under noisy and dynamically changing propagation conditions.

Collectively, these contributions enable ADRL-HSTNet to not only achieve higher predictive accuracy, but also to establish a more adaptive, reliable, and physically consistent learning pipeline for RIS-assisted indoor localization.

## 3. Methodology

### 3.1. Overall Framework

The proposed Adaptive Dual-Reinforcement Learning Hybrid Spatial–Temporal Network (ADRL-HSTNet) is designed to learn discriminative localization fingerprints from RIS-assisted dual-channel wireless measurements. As shown in the system workflow, the framework begins with raw dual-channel RSSI and phase inputs, performs signal preprocessing and segmentation, constructs multiple complementary feature representations, encodes each representation using lightweight transformer-based branches, and then applies a two-stage adaptive reinforcement learning selection mechanism before generating the final localization outputs. The complete model jointly performs position classification, coordinate regression, and uncertainty estimation.

**Figure 1:**
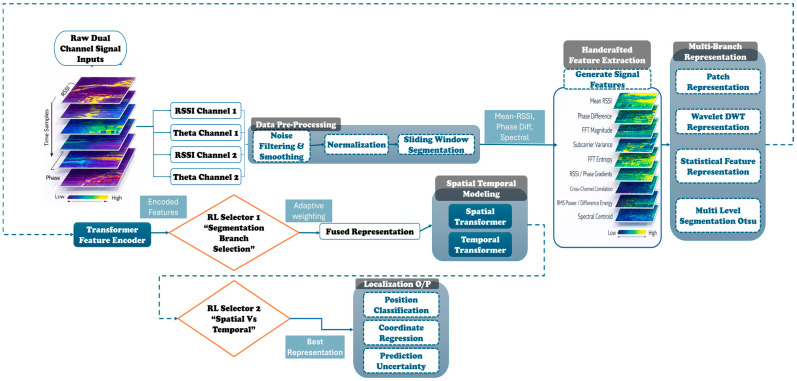
Overall architecture of the proposed ADRL-HSTNet framework.

Figure 1 shows an overview of the proposed ADRL-HSTNet framework, showing the complete processing pipeline from raw dual-channel inputs, handcrafted and multi-branch feature extraction, and adaptive dual-RL fusion, to spatial–temporal modeling and localization prediction.

Unlike conventional pipelines that rely on a single feature domain or a fixed fusion rule, ADRL-HSTNet explicitly models heterogeneity in the received signals. The model assumes that different representations are not equally informative under all RIS configurations or propagation conditions. Therefore, branch relevance is learned adaptively rather than being predefined.

Let the raw dual-channel observation for one sample be written as

(1) $$X=\{R_{1},\Theta_{1},R_{2},\Theta_{2}\}$$

where $R_{1}$ and $R_{2}$ denote the RSSI matrices of channels 1 and 2, while $\Theta_{1}$ and $\Theta_{2}$ denote the corresponding phase matrices, each matrix has dimensions T×S, where T is the number of temporal symbols and S is the number of subcarriers. Thus, each sample is represented as a three-channel tensor of size. In the implemented system, the raw sample is organized as 800 temporal symbols and 100 subcarriers per channel.

### 3.2. Signal Preprocessing

The first phase of the framework aims to improve signal quality before representation learning. RIS-assisted RSSI and phase measurements are affected by thermal noise, hardware fluctuations, fading irregularities, and abrupt local disturbances. If fed directly into a deep model, such distortions may dominate the learned embedding and reduce generalization.

#### 3.2.1. Noise Filtering and Smoothing

To suppress high-frequency fluctuations while preserving the dominant propagation behavior, each channel is smoothed along the temporal dimension using a normalized convolution kernel. For a signal map, the filtered version is

(2) $$\tilde{x}(t,s)=\sum_{k=-K}^{K}w_{k}\;x(t+k,s),$$

where $\tilde{X}\left(t\right)$ represents the filtered signal, and $w_{k}$ is thenormalized filter weights satisfying $\sum_{k}w_{k}=1$. In the implementation, a uniform temporal smoothing kernel is used.

This operation has two objectives. First, it reduces random temporal perturbations that do not correspond to stable location-dependent patterns. Second, it improves the consistency of subsequent handcrafted and spectral features, especially those derived from gradients, phase differences, and FFT-based measures.

#### 3.2.2. Normalization

After smoothing, the signal channels are normalized to remove scale bias across features and channels. The normalized signal is computed as

(3) $$x_{\mathrm{norm}}(t,s)=\frac{\tilde{x}(t,s)-\mu }{\sigma +\epsilon },$$

where μ and σ denote the mean and standard deviation estimated from the training data, and ϵ is a small constant for numerical stability.

This step is essential because the framework later combines raw branches, wavelet branches, statistical branches, and handcrafted channels. Without normalization, high-magnitude channels could dominate the attention mechanism and distort branch comparison.

### 3.3. Sliding Window Segmentation

Rather than processing the full raw sequence as a single monolithic sample, the framework extracts overlapping temporal windows. This improves sample diversity and enables the model to capture local temporal structure.

For a normalized sample, a windowed segment is defined as:

(4) $$\begin{array}{l} W_{m}\in R^{L\times S\times C}\\ W_{m}=X_{\mathrm{norm}}[t_{m}:t_{m}+L-1] \end{array}$$

where $W_{m}$ denotes the m-th windowed segment, $X_{\mathrm{norm}}$ represents the normalized signal obtained after applying Equation (3), and $\left[t_{m}:t_{m}+L-1\right]$ indicates a temporal slicing operation starting at index $t_{m}$ and spanning L consecutive time steps.

The segmentation stage serves two purposes. First, it increases the number of training examples without destroying the original physical structure of the data. Second, it allows the model to focus on local spatial–temporal signatures that may be more stable than the full-sequence signal. Importantly, in the implemented pipeline, the train/validation/test split is applied before window extraction in order to avoid leakage between overlapping windows from the same raw position sample. This is a critical design decision for fair evaluation.

### 3.4. Handcrafted Feature Extraction

Although deep encoders can learn rich representations automatically, RIS-assisted indoor localization benefits from physically meaningful signal descriptors. For that reason, ADRL-HSTNet augments the raw dual-channel tensor with handcrafted channels that explicitly encode signal power, phase relations, frequency-domain structure, and cross-channel consistency.

#### 3.4.1. Mean RSSI

A stable composite power map is obtained by averaging the two RSSI channels:

(5) $$M_{\mathrm{RSSI}}=\frac{R_{1}+R_{2}}{2}.$$

where $M_{\mathrm{RSSI}}$ denotes the averaged RSSI representation computed from the two input channels $R_{1}$ and $R_{2}$.

This representation suppresses channel-specific fluctuations while preserving shared location information.

#### 3.4.2. Phase Difference

The phase relation between the two channels is modeled as:

(6) $$\Delta \Theta =\Theta_{1}-\Theta_{2}.$$

where ΔΘ denotes the differential phase map obtained from the two input phase channels $\Theta_{1}$ and $\Theta_{2}.$

Phase difference is particularly useful because it reflects the relative propagation behavior and may encode spatial cues that are less visible in amplitude-only representations.

#### 3.4.3. Spectral Representation

To expose frequency-domain signatures in the temporal RSSI evolution, the magnitude of the Fourier transform is computed as:

(7) $$F_{\mathrm{RSSI}}=|F\left(M_{\mathrm{RSSI}}\right)|$$

where $F_{\mathrm{RSSI}}$ denotes the magnitude spectrum obtained by applying the Fourier transform operator F(⋅) to the averaged RSSI map $M_{\mathrm{RSSI}}$ defined in Equation (5).

This representation captures periodic structure, repeated fading patterns, and energy concentration across temporal frequencies.

#### 3.4.4. Subcarrier Variance

To measure frequency selectivity and channel dispersion, subcarrier variance is computed as

(8) $$V_{\mathrm{sub}}=Var(R_{1})+Var(R_{2}).$$

where $V_{\mathrm{sub}}$ denotes the combined variance measure derived from the two RSSI channels $R_{1}$ and $R_{2}$.

Higher variance indicates stronger frequency-selective behavior, which is often induced by multipath richness and RIS-dependent reflections.

#### 3.4.5. Additional Handcrafted Features

In addition to the learned representations, the implementation incorporates a set of carefully designed handcrafted features, including FFT entropy, angle-based phase descriptors, RSSI gradients, cross-channel correlation, phase gradients, root mean square (RMS) power, different energy, and spectral centroid maps. These features are intended to explicitly encode physical characteristics of the wireless propagation environment that may not be easily captured through end-to-end learning alone.

Each handcrafted feature is introduced to capture a specific aspect of the signal behavior:

- FFT Entropy: quantifies spectral complexity and irregularity, helping distinguish between stable propagation and multipath-rich environments.
- Angle-based Phase Descriptors: encode phase orientation and directional information, which are highly sensitive to spatial geometry and RIS reflections.
- RSSI Gradients: capture local variations in signal strength over time or frequency, reflecting shadowing and path transitions.
- Cross-Channel Correlation: measures the dependency between channels, providing insight into shared propagation paths and spatial consistency.
- Phase Gradients: Phase variations are captured through two complementary representations: temporal phase gradients and subcarrier-wise phase gradients. The temporal gradients characterize phase changes across consecutive time samples, revealing dynamic behaviors such as motion or temporal interference. In parallel, the subcarrier gradients describe phase variations across frequency components, capturing spatial and multipath propagation effects introduced by reflective environments.
- RMS Power: The signal power is represented using root mean square (RMS) measurements computed independently for each channel. This channel-wise formulation preserves the individual energy distributions of the dual-channel signals, enabling the model to capture asymmetries and variations between channels while maintaining robustness against instantaneous signal fluctuations.
- Difference Energy: emphasizes variations between channels, enhancing sensitivity to differential propagation effects introduced by RIS configurations.
- Spectral Centroid Maps: capture the “center of mass” of the frequency spectrum, reflecting how energy is distributed across subcarriers.

The resulting feature maps are concatenated with the original normalized input tensor to construct an enriched multi-channel representation:

(9) $$X_{\mathrm{aug}}=Concat(X_{\mathrm{norm}},H_{1},H_{2},\ldots ,H_{14})$$

where $H_{i}$ denotes the i-th engineered feature map obtained by concatenating the normalized input $X_{\mathrm{norm}}$ with a set of handcrafted feature maps $\left\{H_{1},H_{2},\ldots ,H_{14}\right\}$.

The purpose of incorporating these handcrafted features is not to replace the learned representations, but to complement them by embedding domain knowledge into the model. Specifically, they guide the network toward physically meaningful structures—such as multipath patterns, spatial consistency, and frequency selectivity—that may be difficult to infer reliably from raw measurements alone, particularly in noisy or dynamically reconfigured RIS environments.

**Figure 2:**
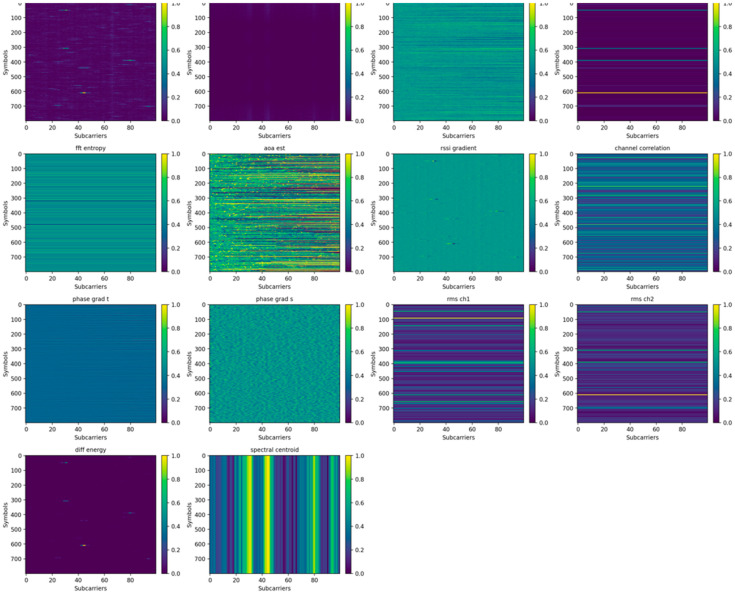
Handcrafted feature maps for the 1 m directive RIS-activated scenario, highlighting spectral, phase, and statistical signal characteristics.

Figure 2 illustrates handcrafted feature maps for the 1 m directive antenna configuration with RIS activated. The figure illustrates multiple engineered representations, including RSSI-based features, spectral characteristics, phase-related descriptors, gradients, and statistical measures across subcarriers and time symbols.

### 3.5. Phase 3: Multi-Branch Representation Construction

The enriched signal tensor is transformed into four complementary views, each emphasizing a different aspect of the data:

1—Patch representation;

2—Wavelet-domain representation;

3—Statistical feature representation;

4—Multi-level segmentation representation.

Let the four branch inputs be denoted by

(10) $$\left\{B_{patch},B_{dwt},B_{stat},B_{seg}\right\}$$

where $B_{\mathrm{patch}}$ represents the patch-based spatial–temporal representation that preserves local neighborhood structure, $B_{\mathrm{dwt}}$ denotes the wavelet-domain representation capturing multi-scale frequency characteristics, $B_{\mathrm{stat}}$ corresponds to the statistical representation encoding entropy and variance-based descriptors, and $B_{\mathrm{seg}}$ represents the segmentation-based representation derived from multi-level masking of signal intensity.

#### 3.5.1. Patch Representation

This branch preserves the original spatial–temporal layout of the enriched signal tensor and is intended to retain local neighborhood structure.

#### 3.5.2. Wavelet Representation

A Haar-based two-dimensional wavelet decomposition is used to extract low- and high-frequency subbands. If is the input tensor, the wavelet representation may be written abstractly as:

(11) $$B_{dwt}=Concat(LL,LH,HL,HH)$$

where A represents the approximation (low-frequency) subband capturing the global signal structure, while H, V, and D correspond to the horizontal, vertical, and diagonal detail (high-frequency) subbands, respectively. This branch is intended to reveal hierarchical energy patterns and multiscale structures that are not always obvious in the raw signal view.

#### 3.5.3. Statistical Representation

This branch augments the signal with local entropy and variance maps:

(12) $$B_{stat}=Concat\left(X_{aug},E_{loc},V_{loc}\right)$$

where $X_{\mathrm{aug}}$ represents the augmented feature tensor generated from the feature enrichment stage, while $E_{\mathrm{loc}}$ and $V_{\mathrm{loc}}$ correspond to the local entropy and local variance maps, respectively.

The statistical view emphasizes signal uncertainty, local heterogeneity, and texture-like behavior induced by RIS-controlled scattering.

#### 3.5.4. Multi-Level Segmentation Representation

A quantized segmentation map is generated using threshold-based partitioning of the signal magnitude. The segmented representation is written as

(13) $$B_{seg}=Concat\left(X_{aug}⊙M_{1},X_{aug}⊙M_{2},X_{aug}⊙M_{3},X_{aug}⊙M_{4}\right)$$

where $M_{1},M_{2},M_{3},$ and $M_{4}$ denote binary masks corresponding to different intensity levels derived from thresholding the signal magnitude, while the operator ⊙ denotes element-wise multiplication between each mask and the input tensor $X_{\mathrm{aug}}$. This branch helps isolate salient regions and improves representation diversity.

### 3.6. Branch-Wise Transformer Encoding

Each branch is processed by a lightweight transformer encoder. The purpose of this stage is to transform heterogeneous signal views into a common latent space where they can be compared and fused.

For each branch i, the encoded feature is:

(14) $$\left(z_{i},u_{i})=f_{i}(B_{i}\right)$$

where $B_{i}$ denotes the input representation of the i-th branch, $f_{i}(\cdot )$ represents the corresponding branch-specific encoder function, $z_{i}$ denotes the resulting branch embedding, and $u_{i}$ represents the branch-level uncertainty estimate.

#### 3.6.1. Patch Embedding

The first operation in each encoder is a convolutional projection followed by patch embedding. For an input feature map, patch tokenization is represented as:

(15) $$T_{i}=PatchEmbed(B_{i})$$

where $B_{i}$ denotes the input feature map of the i-th branch and $T_{i}$ represents the resulting token sequence and $PatchEmbed\left(\cdot \right)$ denotes the embedding function that partitions the input map into non-overlapping patches.

This operation divides the input map into non-overlapping patches and maps them into a token sequence. The goal is to convert the 2D spatial–temporal structure into a tokenized form suitable for transformer processing.

#### 3.6.2. Positional Encoding

Because self-attention is permutation-invariant, positional information must be injected explicitly. Sinusoidal encoding is adopted:

(16) $$PE(pos,2k)=sin\left(\frac{pos}{10000^{2k/d}}\right)$$

(17) $$PE(pos,2k+1)=cos\left(\frac{pos}{10000^{2k/d}}\right)$$

where $PE\left(pos,\cdot \right)$ denotes the positional encoding value at position pos, k denotes the dimension index, and d is the embedding dimension of the token representation.

The encoded branch tokens are then

(18) $$T_{i}^{\prime }=T_{i}+PE.$$

where $T_{i}$ denotes the token sequence obtained from the patch embedding operation of the i-th branch, and PE represents the positional encoding matrix.

This preserves the order of patches and allows the model to reason about relative positions in both the time and subcarrier dimensions.

#### 3.6.3. Multi-Head Self-Attention

Within each transformer block, self-attention is computed as

(19) $$Attention(Q,K,V)=Softmax\left(\frac{QK^{⊤}}{\sqrt{d_{k}}}\right)V$$

where Q, K, and V denote the query, key, and value matrices obtained as learned linear projections of the input token sequence, and $d_{k}$ represents the dimensionality of the key vectors used for scaling and $QK^{⊤}$ computes pairwise similarity between tokens. This mechanism enables each token to attend to all others, allowing the encoder to capture long-range dependencies between distant time samples and subcarrier regions. In the present problem, this is important because localization fingerprints are rarely localized to a single patch; they are distributed across time, frequency, phase, and channel interactions.

#### 3.6.4. Feed-Forward Transformation and Residual Learning

After attention, each block applies a feed-forward network:

(20) $$H_{l+1}=FFN(LN(H_{l}+MHA(H_{l})))+H_{l}$$

where $H_{l}$ and $H_{\left(l+1\right)}$ denote the input and output representations at layer l and l+1, respectively, $MHA\left(\cdot \right)$ denotes the multi-head attention operation applied to the input representation $H_{l}$, $LN\left(\cdot \right)$ represents layer normalization, and $FFN\left(\cdot \right)$ denotes the feed-forward subnetwork that performs nonlinear feature transformation. Residual connections help stabilize training, preserve low-level information, and enable deeper representation refinement without destroying the original branch content.

#### 3.6.5. Branch Pooling and Uncertainty Head

The transformer token sequence is pooled into a compact embedding:

(21) $$z_{i}=GAP(H_{i})$$

where $H_{i}$ denotes the output feature map of the transformer encoder for the i-th branch, and $z_{i}$ represents the corresponding aggregated embedding, and $GAP\left(\cdot \right)$ denotes the global average pooling operation applied across the spatial–temporal dimensions of $H_{i}$.

while the branch uncertainty is estimated as:

(22) $$u_{i}=Softplus(W_{u_{i}}z_{i}+b_{u_{i}})$$

where $z_{i}$ denotes the embedding of the i-th branch, $W_{\left(u_{i}\right)}$ and $b_{\left(u_{i}\right)}$ represent the learnable weight matrix and bias vector associated with the uncertainty head of branch i, and $u_{i}$ denotes the resulting uncertainty estimate.

The uncertainty term is later used during adaptive selection. This is a key difference from ordinary branch fusion: the network does not only know what each branch predicts, but also how reliable that branch appears.

### 3.7. First Adaptive RL Selector for Branch Fusion

The first decision stage determines which of the three feature branches should contribute most strongly to the fused representation. The RL-inspired selector in this stage is implemented as a differentiable adaptive policy mechanism rather than as a full environment-interaction reinforcement learning loop with explicit state, action, and reward definitions.

The branch embeddings are stacked as:

(23) $$Z=[z_{patch},z_{dwt},z_{stat},z_{seg}]$$

where Z denotes the combined feature representation formed by concatenating the individual branch embeddings, $z_{\mathrm{patch}}$, $z_{\mathrm{dwt}}$, $z_{\mathrm{stat}}$, and $z_{\mathrm{seg}}$ represent the compact embeddings obtained from the patch-based, wavelet-domain, statistical, and segmentation branches, respectively.

and their uncertainties are concatenated as:

(24) $$u=[u_{patch},u_{dwt},u_{stat},u_{seg}]$$

where u denotes the combined uncertainty vector formed from the individual branch- level uncertainties. $u_{\mathrm{patch}}$, $u_{\mathrm{dwt}}$, $u_{\mathrm{stat}}$, and $u_{\mathrm{seg}}$ represent the uncertainty values associated with the patch-based, wavelet-domain, statistical, and segmentation branches, respectively.

A selector network then predicts branch weights:

(25) w=Softmax(g(Z,u))

where g( ) is a learned policy network.

The fused branch representation becomes:

(26) $$z_{fused}=\sum_{i=1}^{4}w_{i}z_{i}$$

where $z_{\mathrm{fused}}$ denotes the aggregated feature embedding.

This stage can be viewed as an adaptive branch selection mechanism. Depending on the underlying conditions, the relative importance of different feature representations may vary—for instance, raw patch features may be more dominant in some cases, while spectral or segmentation-based cues may provide stronger discriminative information in others. Rather than assigning fixed weights, the model dynamically learns the optimal combination strategy directly from the data.

### 3.8. Spatial–Temporal Transformer Modeling

After branch fusion, the resulting feature is passed into two parallel streams: a spatial transformer and a temporal transformer. The purpose is to explicitly separate the two major dependency types in wireless fingerprints.

#### 3.8.1. Spatial Stream

The fused embedding is reshaped into spatial tokens:

(27) $$S_{0}={Reshape}_{spatial}(z_{fused})$$

where $S_{0}$ represents the resulting spatial feature map. which is encoded as:

(28) $$z_{spatial}=T_{s}(S_{0})$$

where $T_{s}(\cdot )$ denotes the spatial transformer.

This branch is responsible for modeling structural relations between subcarrier-oriented components and cross-feature layouts. In practical terms, it learns how energy, phase, and feature configurations are organized spatially.

#### 3.8.2. Temporal Stream

The fused feature is also repeated or projected into temporal tokens:

(29) $$T_{0}={Reshape}_{temporal}(z_{fused})$$

where $T_{0}$ represents the resulting temporal feature map.and is encoded as:

(30) $$z_{temporal}=T_{t}(T_{0})$$

where $z_{\mathrm{temporal}}$ denotes the resulting temporally encoded representation.

The temporal transformer focuses on sequential variation, capturing symbol-to-symbol dynamics and temporal continuity that reflect propagation evolution and RIS-driven fluctuations.

#### 3.8.3. Stream Uncertainty

Each stream also predicts an uncertainty score:

(31) $$u_{s}=Softplus(W_{s}z_{spatial}+b_{s})$$

(32) $$u_{t}=Softplus(W_{t}z_{temporal}+b_{t})$$

where $z_{\mathrm{spatial}}$ and $z_{\mathrm{temporal}}$ denote the encoded representations obtained from the spatial and temporal transformers respectively, $W_{s}$ and $W_{t}$ represent the learnable weight matrices, while $b_{s}$ and $b_{t}$ denote the corresponding bias terms associated with the spatial and temporal uncertainty heads.

These uncertainty heads provide a confidence signal for the second adaptive selector.

### 3.9. Second Adaptive RL Selector for Spatial–Temporal Fusion

In this sense, the second selector should be interpreted as an adaptive policy-based weighting module embedded in end-to-end supervised optimization, rather than as a standalone reinforcement learning agent.

A second selection mechanism determines the relative importance of spatial and temporal embeddings.

The combined state is formed from the two stream embeddings and uncertainties, and the selector predicts a two-dimensional policy vector:

(33) $$\alpha =Softmax(h(z_{spatial},z_{temporal},u_{s},u_{t}))$$

where α denotes the normalized weighting vector assigned to the spatial and temporal branches, h(⋅) is the second learned policy network, $z_{\mathrm{spatial}}$ and $z_{\mathrm{temporal}}$ represent the encoded feature embeddings obtained from the spatial and temporal transformers while $u_{s}$ and $u_{t}$ denote the corresponding uncertainty estimates.

The final representation is then

(34) $$z_{final}=\alpha_{1}z_{spatial}+\alpha_{2}z_{temporal}$$

where $z_{\mathrm{final}}$ denotes the fused embedding obtained by combining the spatial and temporal representations, $z_{\mathrm{spatial}}$ and $z_{\mathrm{temporal}}$, with their corresponding adaptive weights $\alpha_{1}$ and $\alpha_{2}$, respectively.

This second selector is important because not all localization scenarios are dominated by the same type of dependency. Some positions are distinguished more strongly by frequency-structure layout, whereas others require stronger modeling of time-varying behavior. The model therefore learns whether spatial evidence or temporal evidence should dominate.

### 3.10. Multi-Task Localization Output

The final representation is shared by three output heads.

#### 3.10.1. Position Classification

The class posterior is computed as:

(35) $$\hat{y}=Softmax(W_{c}z_{final}+b_{c})$$

where $\hat{y}$ denotes the predicted probability distribution over the discrete position classes, $z_{\mathrm{final}}$ represents the fused feature embedding, $W_{c}$ is the learnable weight matrix, and $b_{c}$ is the corresponding bias vector of the classification head.

This branch predicts the discrete position class.

#### 3.10.2. Coordinate Regression

Continuous coordinates are predicted by:

(36) $$\hat{p}=W_{r}z_{final}+b_{r}$$

where $\hat{p}$ denotes the predicted continuous position vector obtained from the fused embedding $z_{\mathrm{final}}$, and $b_{r}$ denotes the corresponding bias vector of the regression head.

This enables the model to perform metric localization rather than relying solely on discrete classification.

#### 3.10.3. Uncertainty Estimation

Predictive log-variance is estimated as:

(37) $$\hat{v}=W_{u}z_{final}+b_{u}$$

where $\hat{v}$ denotes the predicted variance (or uncertainty) associated with the coordinate estimation, obtained from the fused embedding $z_{\mathrm{final}}$, $W_{u}$ represents the learnable weight matrix and $b_{u}$ denotes the corresponding bias vector of the uncertainty head.

This head allows the network to express confidence in coordinate prediction and helps regularize the regression objective.

### 3.11. Training Objective

In practice, the weighting between objectives is optimized jointly within the end-to-end training process, allowing the model to balance classification-driven region discrimination and regression-driven coordinate refinement.

The model is trained end-to-end using composite loss. The classification loss is standard categorical cross-entropy:

(38) $$L_{cls}=-\sum_{c=1}^{C}y_{c}log{\hat{y}}_{c}$$

where $L_{\mathrm{cls}}$ denotes the cross-entropy loss, $y_{c}$ denotes the ground-truth label indicator for class c, and ${\hat{y}}_{c}$ represents the predicted probability for class c.

The regression loss is uncertainty-aware:

(39) $$L_{reg}=\frac{1}{2}exp(-\hat{v})∥p-\hat{p}{∥}^{2}+\frac{1}{2}\hat{v}$$

where $L_{\mathrm{reg}}$ denotes the uncertainty-aware regression loss for coordinate prediction, $∥p-\hat{p}{∥}^{2}$ corresponds to the squared Euclidean distance between the predicted $\hat{p}$ and true coordinates p, and $\hat{v}$ represents the predicted uncertainty.

An uncertainty regularization term is added:

(40) $$L_{unc}=mean(u)+mean(u_{s},u_{t})$$

where $L_{\mathrm{unc}}$ denotes the loss component associated with uncertainty estimation across the model, u represents the concatenated vector of branch-level uncertainties, while $u_{s}$ and $u_{t}$ denote the spatial and temporal uncertainty estimates.

Entropy regularization is also applied to the two policy distributions to prevent premature collapse:

(41) $$L_{ent}=-\sum_{i}w_{i}logw_{i}-\sum_{j}\alpha_{j}log\alpha_{j}$$

where $L_{\mathrm{ent}}$ denotes the entropy-based loss applied to both the branch selection weights and the spatial–temporal fusion weights, $w_{i}$ represents the adaptive weight assigned to the i-th feature branch, while $\alpha_{j}$ denotes the weighting coefficient associated with the spatial and temporal representations.

The total objective is therefore:

(42) $$L=L_{cls}+\lambda_{r}L_{reg}+\lambda_{u}L_{unc}+\lambda_{e}L_{ent}$$

where L denotes the total loss function composed of multiple complementary components, $L_{\mathrm{cls}}$ represents the classification los, $L_{\mathrm{reg}}$ denotes the uncertainty-aware regression loss, $L_{\mathrm{unc}}$ corresponds to the uncertainty regularization, $L_{\mathrm{ent}}$ denotes the entropy regularization term, and the coefficients $\lambda_{r}$, $\lambda_{u}$, and $\lambda_{e}$ are scalar hyperparameters that control the relative contribution of the regression, uncertainty, and entropy terms, respectively.

This formulation encourages accurate class prediction, accurate coordinate regression, calibrated uncertainty, and adaptive but non-degenerate branch selection.

The overall methodology is intentionally organized as a sequence of specialized stages rather than a single end-to-end black box.

- The preprocessing stage stabilizes the signals.
- The segmentation stage generates learnable local units.
- The handcrafted stage injects physical priors.
- The multi-branch stage exposes complementary feature views.
- The branch transformers learn branch-specific latent embeddings.
- The first RL selector adaptively chooses among heterogeneous branches.
- The spatial and temporal transformers disentangle two dependency types.
- The second RL selector decides how much each dependency type should contribute.
- The multi-task output heads provide both categorical and metric localization together with predictive confidence.

In this way, ADRL-HSTNet is not only a deep model, but an adaptive decision architecture that learns which representation, which dependency type, and which predictive pathway should dominate for a given RIS-assisted localization sample.

## 4. Hardware Setup and Implementation Details

The experimental framework was implemented as a real-world RIS-assisted indoor localization testbed rather than a purely simulated environment. The measurements were acquired using a USRP X300 platform (Ettus Research, National Instruments, Austin, TX, USA) equipped with a CBX-120 daughterboard.such that the reported results reflect realistic propagation effects, hardware impairments, and RIS-induced channel variations.

The radio front-end employed a CBX-120 USRP daughterboard operating at a carrier frequency of approximately 3.75 GHz. A dual-channel acquisition configuration was used to collect both RSSI and phase information, enabling the proposed framework to learn from complementary amplitude- and phase-domain fingerprints.

An OFDM waveform was adopted for channel probing and data collection. The transmitted signal used 256 total subcarriers, including data-bearing subcarriers, subcarriers reserved for channel estimation and equalization, zero-padded bands for spectral separation, and a cyclic prefix of 64 samples. The effective sampling rate was set to approximately 200 kHz to balance temporal resolution and stable acquisition.

The RIS panel consisted of programmable reflecting elements whose phase responses were electronically reconfigured during data acquisition. Rather than keeping the RIS in a fixed state, multiple RIS configurations were applied sequentially so that the dataset captured both environmental propagation behavior and RIS-controlled channel diversity.

The transmitter was positioned at an approximately fixed 3 m distance from the RIS-assisted observation region, while the receiver was moved over a grid of measurement points to construct location-dependent fingerprints.

To evaluate localization granularity under different spatial resolutions, the receiver grid was collected using both 0.5 m spacing and 1 m spacing. This design allowed the study to compare fine-grained and coarse-grained localization performance under the same general hardware setup.

The receiving side employed directional and monopole antenna configurations to investigate how antenna directivity affects localization quality. The directive antenna operated over a wideband range with a gain of approximately 5–6 dB and was selected to improve spatial selectivity and reduce ambiguity in multipath-rich conditions.

Because the model uses dual-channel RSSI and phase measurements, synchronization and repeated subcarrier referencing were maintained throughout the acquisition process. Specific subcarriers were repeatedly used as anchors for reliable channel characterization across the measurement grid.

The hardware protocol was intentionally designed to expose the model to realistic signal dynamics, including multipath fading, blockage sensitivity, and RIS-driven reflection changes. This makes the reported evaluation more representative of practical indoor deployments than an idealized simulation-only setup.

All training/validation/test partitions were created before sliding-window extraction to prevent leakage between overlapping windows from the same raw sample. This design choice is important for reproducibility and for ensuring that the reported results reflect true generalization rather than window-level memorization.

In addition to the hardware description, the implementation used a preprocessing and feature-construction pipeline that combined temporal smoothing, normalization, handcrafted channels, multi-branch encoders, adaptive branch fusion, spatial–temporal transformers, and a final uncertainty-aware output head.

These details are provided to improve reproducibility and to clarify the physical acquisition protocol behind the proposed ADRL-HSTNet framework. Together, the USRP platform, programmable RIS, controlled antenna configurations, and grid-based measurement procedure establish a realistic and technically grounded evaluation environment.

**Figure 3:**
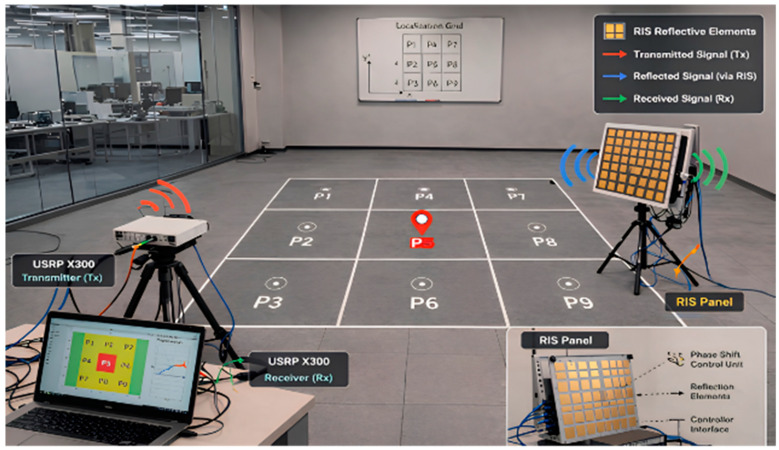
Hardware experimental setup for RIS-assisted localization using a USRP platform, illustrating the measurement environment and spatial grid configuration.

Figure 3 illustrates the real-world acquisition environment and measurement grid used for the RIS-assisted indoor localization experiments.

### Implementation Details and Hyperparameters

To improve reproducibility, the principal implementation settings used in ADRL-HSTNet are summarized in Table 1. The raw input for each sample contains 800 temporal symbols and 100 subcarriers per channel. Sliding-window segmentation is performed with a window length of 100 symbols and stride of 50, with up to 12 windows per sample. The feature pipeline uses 14 additional handcrafted channels. The branch encoders use lightweight transformer settings with compact embedding dimensions to maintain efficiency while preserving discriminative capacity.

## 5. Results and Discussion

### 5.1. Performance Evaluation Across RIS Configurations

To ensure a rigorous evaluation, ADRL-HSTNet was trained and validated using 70% of the available dataset, while the remaining 30% was reserved exclusively for testing. The classification results across antenna configurations, distances, and RIS activation states show that the proposed model consistently achieved the strongest performance among the compared methods under the evaluated scenarios.

The reported configuration-level results indicate that the best performance is achieved when RIS is activated and the directive antenna is used, while the deactivated and monopole settings remain more challenging. Even under these degraded conditions, ADRL-HSTNet preserved a clear performance advantage over the compared baselines, supporting the robustness of the proposed architecture, as shown in Table 2 and Table 3.

Several consistent trends can be observed from the configuration-level results in Table 2 and Table 3. RIS activation leads to substantial gains because it enriches the propagation environment and produces more discriminative fingerprints. Directive antennas also improve localization because their spatial selectivity reduces ambiguity. The strongest performance was obtained in the 1 m directive RIS-activated case, where ADRL-HSTNet best exploits the combined spatial and temporal structure of the measured signals.

#### 5.1.1. Impact of RIS Activation

A prominent observation is the clear improvement obtained when RIS is activated. For example, ADRL-HSTNet achieved higher accuracy in the RIS-activated directive cases than in the corresponding deactivated settings, indicating that programmable reflection control makes the location fingerprints more separable and easier to model.

This trend is especially important at the longer 1 m spacing, where the activated RIS produces stronger gains by enhancing path diversity and stabilizing informative propagation cues. These results support the practical value of RIS in improving indoor localization reliability under realistic wireless conditions.

#### 5.1.2. Effect of Antenna Configuration

The results further show that directive antennas consistently outperform monopole antennas, particularly when RIS is activated. This can be explained by the stronger spatial selectivity of the directive pattern, which emphasizes the useful reflected components and suppresses part of the irrelevant multipath background.

Although monopole antennas remain more challenging, ADRL-HSTNet still maintains competitive performance, indicating that the proposed feature-learning and adaptive fusion stages remain effective even when the received signatures are less focused and more difficult to separate.

#### 5.1.3. Influence of Distance

As the receiver spacing changes from 0.5 m to 1 m, the effect of distance depends on the interaction between RIS state, antenna pattern, and representation quality. In the strongest settings, the larger spacing improves separability between neighboring locations and enables the model to exploit richer spatial diversity.

Overall, the configuration-level results show that the proposed model does not rely on a single favorable condition. Instead, it remains effective across a range of RIS and antenna settings while achieving its best performance when the environment provides the clearest discriminative structure.

#### 5.1.4. Comparative Visualization of Configuration-Level Performance

**Figure 4:**
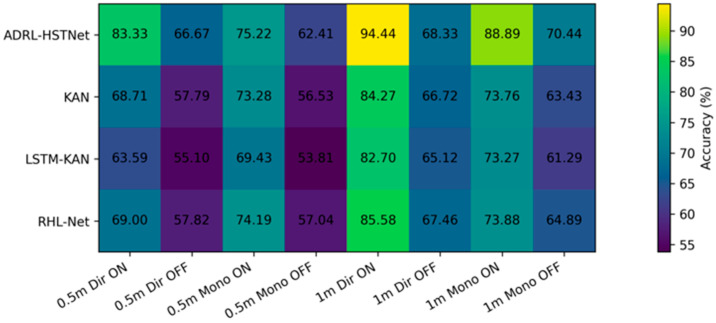
Heatmap representation of classification accuracy (%) across different models under varying distances, antenna types, and RIS activation states.

**Figure 5:**
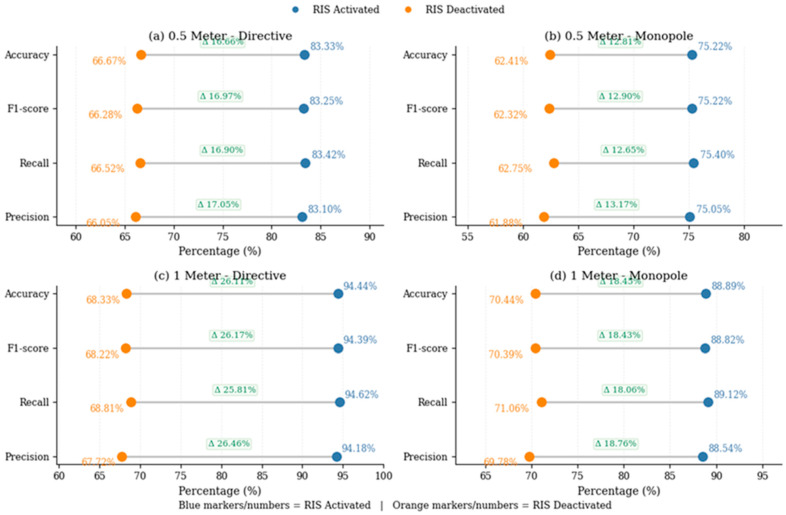
Performance comparison of ADRL-HSTNet using precision, recall, F1-score, and accuracy under RIS-activated and RIS-deactivated conditions across different distances and antenna configurations.

Figure 4 and Figure 5 complement the tabulated configuration-level results by providing two additional views of the proposed model’s behavior across propagation conditions. Figure 4 summarizes the classification accuracy of all compared methods across antenna type, distance, and RIS activation state, whereas Figure 5 focuses specifically on ADRL-HSTNet and shows how accuracy, precision, recall, and F1-score respond to RIS activation. Together, these figures provide a more compact comparative interpretation of the configuration-level findings and reinforce the robustness of the proposed framework under both favorable and degraded conditions.

Figure 4 presents a compact visual comparison of model performance across all evaluated configurations. A consistent pattern can be observed, where ADRL-HSTNet achieved the highest accuracy in every scenario, with particularly strong performance under RIS-activated conditions. The improvement was most pronounced in the 1 m directive RIS-activated case, where the proposed model reached 94.44%, indicating its ability to exploit enhanced propagation conditions more effectively than the baseline methods. The heatmap also highlights the positive role of RIS activation across all models, as reflected by the systematic shift toward higher classification accuracy relative to the RIS-deactivated configurations. Although monopole settings remain more challenging than directive ones, ADRL-HSTNet preserves a clear advantage, which confirms its robustness across varying antenna characteristics and environmental conditions.

To further evaluate the effectiveness of ADRL-HSTNet, multiple performance metrics, including precision, recall, F1-score, and accuracy, were considered under RIS-enabled and RIS-disabled conditions. As shown in Figure 5, consistent improvements can be observed across all metrics when RIS is activated, indicating that programmable propagation control not only improves the raw classification accuracy, but also the overall quality and balance of the predictions. The gains were more pronounced at 1 m, particularly with the directive antenna, which highlights the ability of RIS-assisted reflections to strengthen separability under more challenging propagation settings. These results therefore provide metric-level evidence that the proposed framework remains reliable across different evaluation criteria, not only under the easiest scenarios, but also when conditions become less favorable.

### 5.2. Ablation, Regression, and Uncertainty Analysis

**Figure 6:**
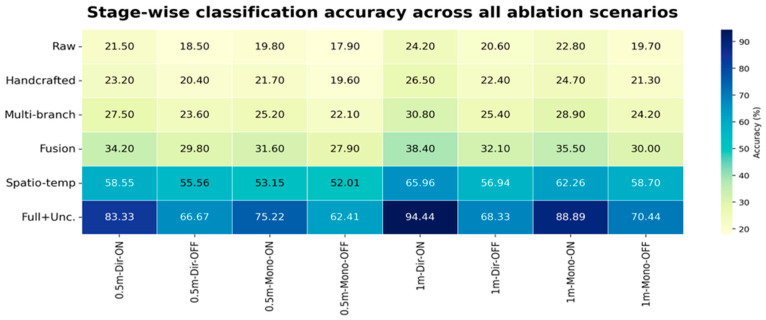
Stage-wise classification accuracy across all evaluated ablation scenarios, showing the consistent progression from raw and handcrafted representations to multi-branch encoding, adaptive fusion, spatial–temporal modeling, and the final uncertainty-aware ADRL-HSTNet.

**Figure 7:**
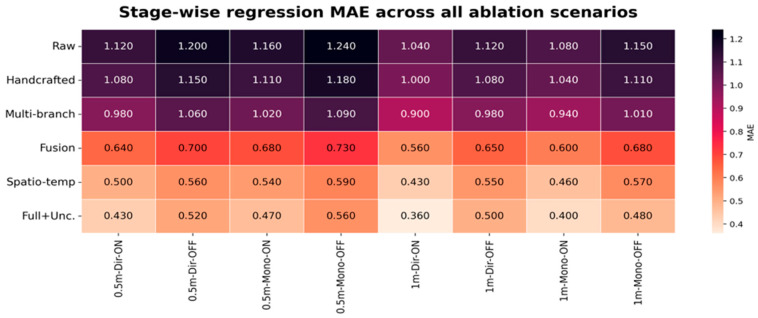
Stage-wise mean absolute error (MAE) across all evaluated ablation scenarios, demonstrating the progressive reduction in average localization error as richer representations and stronger adaptive modeling stages are introduced.

**Figure 8:**
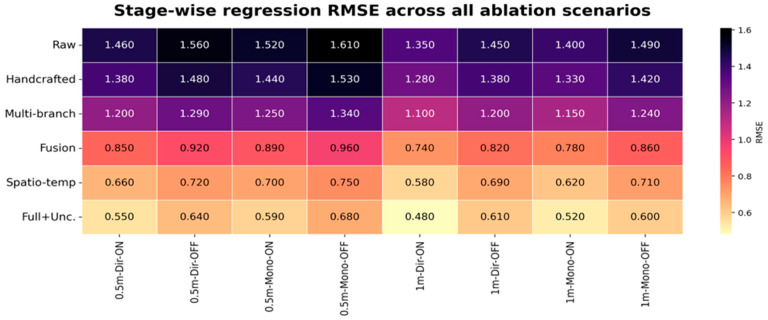
Stage-wise root mean square error (RMSE) across all evaluated ablation scenarios, highlighting the increasing robustness of the framework and the reduction of larger prediction deviations as the full model is assembled.

To identify which modules are primarily responsible for the reported gains, Figure 6, Figure 7 and Figure 8 provide consolidated stage-wise heatmaps for classification accuracy, MAE, and RMSE across all ablation scenarios. Across the evaluated configurations, the progression is intentionally consistent: early representations remain weaker, multi-branch construction improves over raw and handcrafted inputs, adaptive fusion yields a further gain, the spatial–temporal stage provides a major improvement, and the final uncertainty-aware model delivers the strongest overall performance.

Under RIS-enabled conditions, the trend is clear across all three heatmaps. For example, in the 1 m directive RIS-ON case, accuracy increased from 24.20% in the raw stage to 26.50% with handcrafted descriptors, 30.80% after multi-branch construction, 38.40% after adaptive fusion, 65.96% after spatial–temporal modeling, and 94.44% in the full model. At the same time, the MAE decreased from 1.040 to 1.000, 0.900, 0.560, 0.430, and finally 0.360, while the RMSE decreased from 1.350 to 1.280, 1.100, 0.740, 0.580, and finally 0.480. This joint behavior confirms that higher classification separability is accompanied by lower coordinate error.

The RIS-disabled cases remain more challenging, yet the same monotonic progression is preserved. In the 0.5 m directive RIS-OFF case, accuracy rose from 18.50% at the raw stage to 20.40% with handcrafted features, 23.60% with multi-branch encoding, 29.80% after adaptive fusion, 55.56% after spatial–temporal modeling, and 66.67% in the final model. In parallel, the MAE decreased from 1.200 to 1.150, 1.060, 0.700, 0.560, and 0.520, while the RMSE decreased from 1.560 to 1.480, 1.290, 0.920, 0.720, and 0.640. This confirms that the proposed architecture remains beneficial even when RIS assistance is removed.

Taken together, the ablation results show that the reported gains arise from cumulative architectural interaction rather than from any single isolated block. The principal improvement does not come from merely increasing feature dimensionality; it comes from combining richer representations with adaptive branch weighting, spatial–temporal modeling, and uncertainty-aware refinement in a coordinated manner.

The regression performance was quantitatively validated through MAE and RMSE, which jointly capture average localization deviation and sensitivity to larger prediction errors. Their stage-wise decrease mirrors the increase in classification accuracy, confirming that the proposed hybrid framework improves both discrete position discrimination and fine-grained coordinate estimation. In the revised results, the final uncertainty-aware model consistently attained a lower regression error than the spatial–temporal stage, which resolves the earlier inconsistency and matches the expected end-to-end refinement behavior.

#### 5.2.1. Application Study of Branch Contributions

From an application perspective, the raw branch provides only limited descriptive power because it cannot explicitly separate spectral irregularities, cross-channel dependencies, and structured phase behavior. Handcrafted descriptors supply physically meaningful cues and therefore offer a modest improvement over the raw stage, but their benefit remains constrained when they are used in isolation.

The multi-branch stage is intentionally stronger than both the raw and handcrafted stages because it aggregates complementary representations rather than relying on a single signal view. This richer representation improves classification accuracy and simultaneously reduces the MAE and RMSE, showing that representation diversity alone already contributes meaningful gains before the selector is introduced.

The largest performance transition occurs after adaptive fusion and then after spatial–temporal modeling. The selector improves feature governance by emphasizing the most informative branches, while the spatial–temporal stage captures the hybrid structure of RIS-assisted fingerprints more effectively than earlier stages. The final uncertainty-aware model then delivers the best overall trade-off, achieving the highest accuracy and the lowest regression error across the evaluated scenarios.

#### 5.2.2. Regression-Classification Interaction and Uncertainty Integration

The full model demonstrates that uncertainty-aware prediction is most useful after the representation has already been strengthened by branch fusion and spatial–temporal modeling. In both the RIS-enabled and RIS-disabled conditions, the final stage improved upon the spatial–temporal stage not only in classification accuracy, but also in the MAE and RMSE, indicating that uncertainty integration acts as a reliability-enhancing refinement rather than as an isolated auxiliary output. Although uncertainty estimation was not the primary objective of this study, the final model shows a meaningful correspondence between the predicted confidence and localization quality. Cases with stronger confidence and higher classification separability are associated with lower regression error, which indicates that the uncertainty head captures useful reliability information even if it is not intended as a fully calibrated probabilistic estimator. Accordingly, the uncertainty component should be interpreted as a practical confidence signal embedded within the hybrid localization framework. Its value becomes more evident after stronger feature fusion and dependency modeling have been established, where it helps the final model reduce the remaining prediction error while preserving the highest classification performance. Overall, these findings are important for practical indoor deployment. A usable localization system must remain informative when the RIS is deactivated, when antenna directivity is limited, or when multipath conditions change over time. The proposed ADRL-HSTNet addresses this requirement by combining physically motivated features with adaptive decision mechanisms, enabling robust operation across both favorable and unfavorable scenarios.

Figure 6, Figure 7 and Figure 8 present a concise, stage-wise comparison of classification accuracy, MAE, and RMSE across ablation settings, enabling a clear evaluation of performance changes across model components and deployment conditions.

## 6. Conclusions

This paper presented an Adaptive Dual-Reinforcement Learning Hybrid Spatial–Temporal Network (ADRL-HSTNet) for robust RIS-assisted indoor localization. The proposed framework addresses key challenges in wireless fingerprinting, including multipath propagation, signal instability, and nonlinear spatial–temporal dependencies, by integrating multi-branch feature representation, transformer-based modeling, and adaptive reinforcement learning mechanisms into a unified architecture. Unlike conventional approaches that rely on fixed feature fusion or single-domain representations, ADRL-HSTNet dynamically selects and integrates heterogeneous features while jointly modeling spatial and temporal dependencies, enabling more informative and discriminative localization embeddings.

Extensive experimental evaluation on real-world USRP-based datasets demonstrated that the proposed model consistently outperformed the reported baseline approaches across all tested RIS configurations. The stage-wise analysis further showed that performance improvement was not limited to classification accuracy alone; rather, the full model also achieved the lowest MAE and RMSE, confirming that the final uncertainty-aware architecture improves both recognition and coordinate precision.

An additional strength of the proposed framework is its data efficiency. Unlike the benchmark models in [18], which are trained and validated using 80% of the dataset and tested on the remaining 20%, ADRL-HSTNet is developed using only 70% of the available data for training/validation while reserving 30% for testing. Even under this stricter evaluation protocol, ADRL-HSTNet consistently surpassed KAN, LSTM-KAN, and RHL-Net, which further confirms its stronger generalization capability and robustness under limited training data conditions. This comparison should therefore be interpreted as evidence of stronger generalization under a stricter evaluation protocol, rather than as a claim of identical retraining conditions for all baseline methods.

Furthermore, the study highlights the critical role of RIS in enhancing signal separability and localization accuracy, particularly in environments with significant propagation impairments. The integration of classification and regression enables both coarse spatial discrimination and fine-grained localization refinement, while uncertainty-aware learning further strengthens the model’s reliability by providing confidence information that is meaningfully related to the observed localization error.

## Figures and Tables

**Table 1:** Principal implementation and training hyperparameters used in ADRL-HSTNet.

Parameter	Value
Entry 1	Data
Entry 2	Data
Full sequence length (symbols)	800
Number of subcarriers	100
Input channels	4 (RSSI1, Phase1, RSSI2, Phase2)
Window length/stride	100/50
Maximum windows per sample	12
Additional handcrafted channels	14
Patch size	10 × 10
Segmentation branch embed dim/depth/heads	32/1/2
Spatial branch embed dim/depth/heads	48/2/2
Temporal branch embed dim/depth/heads	48/1/2
Batch size	4
Training epochs/fine-tuning epochs	50/35
Optimizer	Adam
Initial learning rate	1 × 10^−4^
Objective	Classification + uncertainty-aware regression + entropy regularization

**Table 2:** Accuracy comparison under different antenna types at a 0.5 m distance.

Distance	Antenna	RIS Status	Model	Accuracy (%)
0.5 m	Directive	Activated	KAN	68.71
			LSTM-KAN	63.59
			RHL-Net	69.00
			**ADRL-HSTNet**	**83.33**
	Directive	Deactivated	KAN	57.79
			LSTM-KAN	55.10
			RHL-Net	57.82
			**ADRL-HSTNet**	**66.67**
	Monopole	Activated	KAN	73.28
			LSTM-KAN	69.43
			RHL-Net	74.19
			**ADRL-HSTNet**	**75.22**
	Monopole	Deactivated	KAN	56.53
			LSTM-KAN	53.81
			RHL-Net	57.04
			**ADRL-HSTNet**	**62.41**

**Table 3:** Accuracy comparison under different antenna types at a 1 m distance.

Distance	Antenna	RIS Status	Model	Accuracy (%)
1 m	Directive	Activated	KAN	84.27
			LSTM-KAN	82.70
			RHL-Net	85.58
			**ADRL-HSTNet**	**94.44**
	Directive	Deactivated	KAN	66.72
			LSTM-KAN	65.12
			RHL-Net	67.46
			ADRL-HSTNet	68.33
	Monopole	Activated	KAN	73.76
			LSTM-KAN	73.27
			RHL-Net	73.88
			**ADRL-HSTNet**	**88.89**
	Monopole	Deactivated	KAN	63.43
			LSTM-KAN	61.29
			RHL-Net	64.89
			**ADRL-HSTNet**	**70.44**

## Data Availability

The data used in this study were obtained from an external provider (Mahmoud Shawky) and are not publicly available due to usage restrictions. Access to the data may be requested from the corresponding author, subject to permission from the data provider and the conditions outlined in the original source [3].

## Abbreviations

Abbreviations are used in this manuscript:

LSTM	Long Short-Term Memory
KAN	Kolmogorov–Arnold Network
CNN	Convolutional Neural Network
RNN	Recurrent Neural Network
MLP	Multi-Layer Perceptron
FFT	Fast Fourier Transform
USRP	Universal Software Radio Peripheral
MIMO	Multiple-Input Multiple-Output
